# How to deal with COVID-19 vaccine hesitancy in pregnant and breastfeeding women? A meta-analysis

**DOI:** 10.1093/eurpub/ckac129.665

**Published:** 2022-10-25

**Authors:** FP Bianchi, P Stefanizzi, MC Di Gioia, N Brescia, S Lattanzio, S Tafuri

**Affiliations:** Interdisciplinary Department of Medicine, University of Study of Bari, Bari, Italy

## Abstract

**Background:**

Pregnant and breastfeeding women are at an increased risk of severe illness from COVID-19 compared to people who are not pregnant. Therefore, the CDC recommends COVID-19 vaccination for women who are pregnant, breastfeeding, and trying to become pregnant or who may become pregnant in the future. Despite this, low vaccination coverages are reported in this population sub-group. The purpose of this study is to estimate the proportion of pregnant and breastfeeding women expressing hesitation to the COVID-19 vaccine worldwide. Determinants of vaccine compliance and options suggestedto address vaccine hesitancy were also analyzed.

**Methods:**

Forty-six studies were included in the meta-analysis and systematic review, selected from scientific articles available in the MEDLINE/PubMed, Google Scholar, and Scopus databases between January 1, 2020 and February 6, 2022. The following terms were used for the search strategy: (adherence OR hesitancy OR compliance OR attitude) AND (covid* OR SARS*) AND (vaccin* OR immun*) AND (pregnan* OR post-partum OR breastfeeding OR lactating).

**Results:**

The vaccine hesitation rate was 48.4% (95%CI=43.4-53.4%). In a sub analysis by study period, the pooled prevalence of vaccine hesitation was 40.0% (95%CI=31.6-46.6%) considering surveys administered in 2020, 58.0% (95%CI=48.9-66.9%) considering surveys administered in the first semester of 2021, and 38.1% (95%CI=25.9-51.2%) considering surveys administered in the second semester of 2021. The main reasons for vaccine hesitation were lack of information about vaccination, opinion that the vaccine is unsafe, and fear of adverse events for both mother and fetus/child.

**Conclusions:**

In order to achieve high vaccination coverage, a multifactorial approach is needed, requiring major social, scientific, and health efforts. The success of the vaccination campaign in this population depends on the capillarity and consistency of the interventions implemented.

**Key messages:**

• Vaccine hesitancy can be a determining factor in the success (or otherwise) of the anti-COVID-19 immunization campaign.

• Vaccine hesitancy in pregnant and breastfeeding women is a genuine public health concern worldwide.

